# Normal Range of Hepatic Fat Fraction on Dual- and Triple-Echo Fat Quantification MR in Children

**DOI:** 10.1371/journal.pone.0117480

**Published:** 2015-02-06

**Authors:** Hyun Joo Shin, Hyun Gi Kim, Myung-Joon Kim, Hong Koh, Ha Yan Kim, Yun Ho Roh, Mi-Jung Lee

**Affiliations:** 1 Department of Radiology and Research Institute of Radiological Science, Severance Children’s Hospital, Yonsei University College of Medicine, Seoul, Korea; 2 Department of Pediatrics, Severance Children’s Hospital, Yonsei University College of Medicine, Seoul, Korea; 3 Biostatistics Collaboration Unit, Yonsei University College of Medicine, Seoul, Korea; The Chinese University of Hong Kong, HONG KONG

## Abstract

**Objectives:**

To evaluate hepatic fat fraction on dual- and triple-echo gradient-recalled echo MRI sequences in healthy children.

**Materials and Methods:**

We retrospectively reviewed the records of children in a medical check-up clinic from May 2012 to November 2013. We excluded children with abnormal laboratory findings or those who were overweight. Hepatic fat fraction was measured on dual- and triple-echo sequences using 3T MRI. We compared fat fractions using the Wilcoxon signed rank test and the Bland-Altman 95% limits of agreement. The correlation between fat fractions and clinical and laboratory findings was evaluated using Spearman’s correlation test, and the cut-off values of fat fractions for diagnosing fatty liver were obtained from reference intervals.

**Results:**

In 54 children (M:F = 26:28; 5–15 years; mean 9 years), the dual fat fraction (0.1–8.0%; median 1.6%) was not different from the triple fat fraction (0.4–6.5%; median 2.7%) (p = 0.010). The dual- and triple-echo fat fractions showed good agreement using a Bland-Altman plot (-0.6 ± 2.8%). Eight children (14.8%) on dual-echo sequences and six (11.1%) on triple-echo sequences had greater than 5% fat fraction. From these children, six out of eight children on dual-echo sequences and four out of six children on triple-echo sequences had a 5–6% hepatic fat fraction. When using a cut-off value of a 6% fat fraction derived from a reference interval, only 3.7% of children were diagnosed with fatty liver. There was no significant correlation between clinical and laboratory findings with dual and triple-echo fat fractions.

**Conclusions:**

Dual fat fraction was not different from triple fat fraction. We suggest a cut-off value of a 6% fat fraction is more appropriate for diagnosing fatty liver on both dual- and triple-echo sequences in children.

## Introduction

Fatty liver is a spectrum of conditions in which hepatocyte triglycerides are increased [1]. In the United States, the prevalence of fatty liver is 25–35% for the general population and 80–90% for obese patients (body mass index [BMI]) > 30 kg/m^2^), [1,2]. In particular, nonalcoholic fatty liver disease (NAFLD) is increasing in children, affecting approximately 3–17% of American children and 38% of overweight or obese children [3,4]. NAFLD includes a wide spectrum of conditions, ranging from simple steatosis to nonalcoholic steatohepatitis, which can progress to cirrhosis [3,5]. Obesity is the most common cause of fatty liver. However, many other conditions such as drugs, storage diseases, chronic viral infections, toxins, and others can lead to fatty change of the liver [1,6]. An early diagnosis of fatty liver can prevent more serious complications by initiating early treatment. This is especially important as fatty liver can be reversed to a normal range if appropriate and prompt corrections are implemented [3,7]. Therefore, early detection of fatty liver and quantification of hepatic fat can be meaningful in many ways.

Ultrasonography can be a useful and easy diagnostic method for detecting fatty liver without any radiation exposure. Although one study attempted to quantitatively analyze hepatic steatosis with ultrasonography [8], available evidence does not support the use of ultrasonography for the diagnosis or grading of fatty liver in children [1,9]. Computed tomography (CT) can detect fat infiltration in the liver by analyzing the attenuation of the liver parenchyma; however, the risk of radiation exposure is a major disadvantage, especially in pediatric patients. In addition, various hepatic conditions such as cirrhosis and inflammation can influence the density of liver parenchyma on CT images [1]. Liver parenchyma biopsy is the gold standard for diagnosing fatty liver. However, this procedure only obtains a small portion of the liver, which can cause sampling bias in addition to being an invasive method requiring sedation in young children [10].

Magnetic resonance imaging (MRI) can detect hepatic fat infiltration simply and accurately. Proton magnetic resonance spectroscopy (MRS) makes it possible to quantitatively measure even small amount of fat content in the liver [1,7]; however, parameter setting and post-processing techniques are not easy [11]. Quantitative measurement of fat fraction using a chemical shift technique, which distinguishes the differences of resonant frequencies between fat and water, can be an easy way to evaluate fat in the liver [12]. It can be obtained using dual- or triple-echo gradient-recalled echo sequences and is not influenced by underlying hepatic conditions such as fibrosis, even in children [5]. These sequences are easy to perform with any scanner from a variety of vendors in a relatively short time.

Fatty liver can be diagnosed histopathologically if the percentage of triglyceride content exceeds 5%, according to the Nonalcoholic Steatohepatitis Clinical Research Network scoring system [13,14]. Previous studies demonstrated that the normal hepatic fat content using fat quantification MRI was less than 5% in adults [1,15]; however, the normal range of hepatic fat fraction using dual- and triple-echo sequences in children is not yet defined to our knowledge.

Therefore, the purpose of this study was to evaluate the normal ranges of hepatic fat fraction on dual- and triple-echo gradient recalled-echo sequences of MRI in healthy children, and suggest a cut-off value for diagnosing fatty liver on both dual- and triple-echo sequences in children.

## Materials and Methods

### Subjects characteristics

The Institutional Review Board of Severance Hospital approved this retrospective study and required neither patient approval nor informed consent for review of patients’ images and medical records. The abdominal MRI examination was routinely performed in the medical check-up clinic at our tertiary hospital, as well as laboratory tests. We reviewed the medical records of children who visited our medical check-up clinic from May 2012 to November 2013. Age, sex, height, weight, BMI, and routine laboratory findings, including liver function tests (aspartate aminotransferase [AST], alanine aminotransferase [ALT]), glucose, cholesterol, albumin, alkaline phosphatase (ALP), total bilirubin, triglycerides, and ferritin levels, obtained on the same day as abdominal MRI examination were reviewed.

We excluded children with abnormal laboratory findings or those who were overweight (BMI greater than 25 kg/m^2^), which can influence hepatic fat infiltration. Abnormal laboratory levels included a serum AST level greater than 34 IU/L, ALT greater than 46 IU/L, glucose greater than 110 mg/dL, cholesterol greater than 220 mg/dL, albumin greater than 5.4 g/dL, ALP greater than 341 IU/L, total bilirubin greater than 1.2 mg/dL, triglycerides greater than 166 mg/dL, and ferritin greater than 336.2 ng/mL.

### MRI acquisition and image analysis

Hepatic fat fraction (%) was measured on dual- and triple-echo gradient-recalledecho sequences of a routine check-up abdominal MR protocol performed on a 3T MR system (Tim Trio; Siemens Medical Solutions, Erlangen, Germany) with a body coil.

For dual-echo chemical-shift gradient-echo MRI (DE-MRI), we obtained axial images of the liver using gradient echo T1-weighted, dual-echo, in-phase and opposed-phase sequences (TR, 226 msec; TE, 1.23 [opposed-phase] /2.46 [in-phase] msec; flip angle, 65°; section thickness, 6 mm; matrix size, 192×256; acquisition time, 14 sec; and field of view, 300×400 cm). For triple-echo MRI (TE-MRI), the imaging protocol included a breath-hold low-flip-angle T1-weighted, triple-echo, spoiled gradient-echo sequence (TR 226 msec; TE 2.46 [in-phase 1]/3.69 [opposed-phase] /4.92 [in-phase 2] msec; flip angle, 20°; section thickness, 6 mm; matrix size, 256 × 192; acquisition time, 19 sec; and field of view, 315×420 cm).

One radiologist (M.J.L.) with 10 years of experience in pediatric radiology randomly drew three regions of interest (ROIs) in each image of homogenous parenchyma of the right hepatic lobe avoiding hepatic vessels on a picture archiving and communication system (Centricity, General Electric Corporation, Milwaukee, WI) and used the mean value of the three signal intensities. The reviewer was blinded to the demographics and clinical and laboratory data of the children at the time of image analysis. The signal intensities were obtained by drawing ROIs at the same location of the liver in both in-phase (IP) and opposed-phase (OP) images. The hepatic fat fraction in DE-MRI was calculated from the equation as follows: dual-echo fat fraction (%) = [(IP-OP)/(2 × IP)] × 100 [7].

Theoretically, three mean values of the signal intensities should be obtained for measuring the triple-echo fat fraction in all of the in-phase 1 (IP1), opposed-phase (OP), and in-phase 2 (IP2) images. The hepatic fat fraction in TE-MRI can be calculated from the equation as follows: triple-echo fat fraction (%) = [{(IP1+IP2)/2—OP}/(IP1+IP2)] × 100 [11]. In our workstation, the triple-echo fat fraction was automatically calculated and we obtained a triple-echo fat map for direct fat fraction measurement by drawing ROIs in this map.

### Statistical analysis

The Wilcoxon signed rank test was used to compare dual- and triple-echo fat fractions after testing for normality. Bland-Altman 95% limits of agreement were used to evaluate the agreement among dual- and triple-echo fat fractions. The Bland-Altman plot was represented by assigning the mean of the dual- and triple-echo fat fractions as the x-axis value and the difference between these two values as the y-axis value. Spearman’s correlation test was used to evaluate the correlation between fat fraction and clinical or laboratory findings. Among the laboratory findings, we evaluated the correlation with serum glucose, cholesterol levels, and triglyceride levels according to previous studies [5,16,17]. *P* values < 0.05 were considered statistically significant for all analyses. Analysis was performed using SPSS version 20.0.0 (IBM Corp., Armonk, NY, USA). The software R version 3.0.1 (The R Foundation for Statistical Computing, Vienna, Austria) was also used for the evaluation of cut-off values of fat fractions using the reference interval and confidence interval, which refer to the variation of the measurements in healthy individuals according to the previously reported guidelines [18].

## Results

### Clinical and laboratory findings

During the study period, a total of 72 children ranging from 5 to 16 years old visited our medical check-up clinic. Among these children, 18 were excluded due to abnormal laboratory findings in 17 children, overweight body habitus in 5 children, and both problems in 4 children. In total, 54 children were enrolled in this study, including 26 boys and 28 girls. The age range was 5–15 years with a mean of 9 years. The mean height was 133.9 ± 17.3 cm with a range of 108–178 cm. The mean weight was 32.0 ±12.4 kg with a range of 16–65 kg. The mean BMI was 17.2 ± 3.0 kg/m^2^ with a range of 12.2–24.3kg/m^2^.

On laboratory findings, the mean AST level was 22.2 ± 4.5 IU/L with a range of 12–32 IU/L and the mean ALT level was 12.2 ± 6.3 IU/L with a range of 6–45 IU/L. The mean glucose level was 90.6 ± 6.5 mg/dL with a range of 74–108 mg/dL. The mean cholesterol level was 167.5 ± 19.6 mg/dL with a range of 122–206 mg/dL. The mean albumin level was 4.5 ± 0.2 g/dL with a range of 4–5 g/dL. The mean ALP level was 180.0 ± 54.9 IU/L with a range of 56–335 IU/L, and the mean total bilirubin level was 0.6 ± 0.2 mg/dL with a range of 0.2–1.2 mg/dL. The mean triglyceride level was 68.5 ± 24.8 mg/dL with a range of 36–148 mg/dL, and the mean ferritin level was 34.9 ± 20.3 ng/mL with a range of 12–111.6 ng/mL.

### Fat fraction on MR

**Fig 1:**
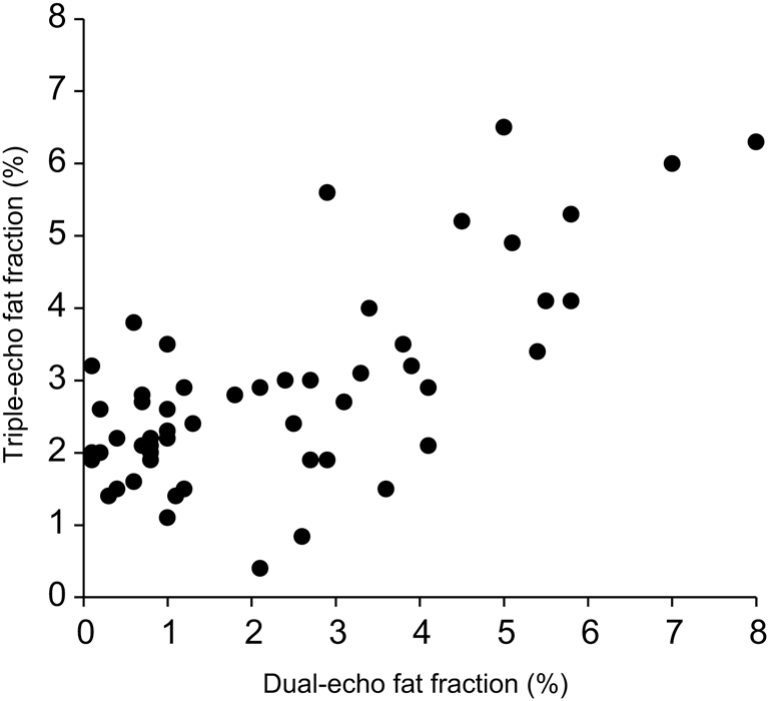
Comparison of hepatic fat fraction (%) in dual- and triple-echo MRI. The dual-echo fat fraction (mean 2.3 ± 2.0%) was not different from the triple-echo fat fraction (mean 2.9 ± 1.4%) in healthy children (*p* = 0.010).

**Fig 2:**
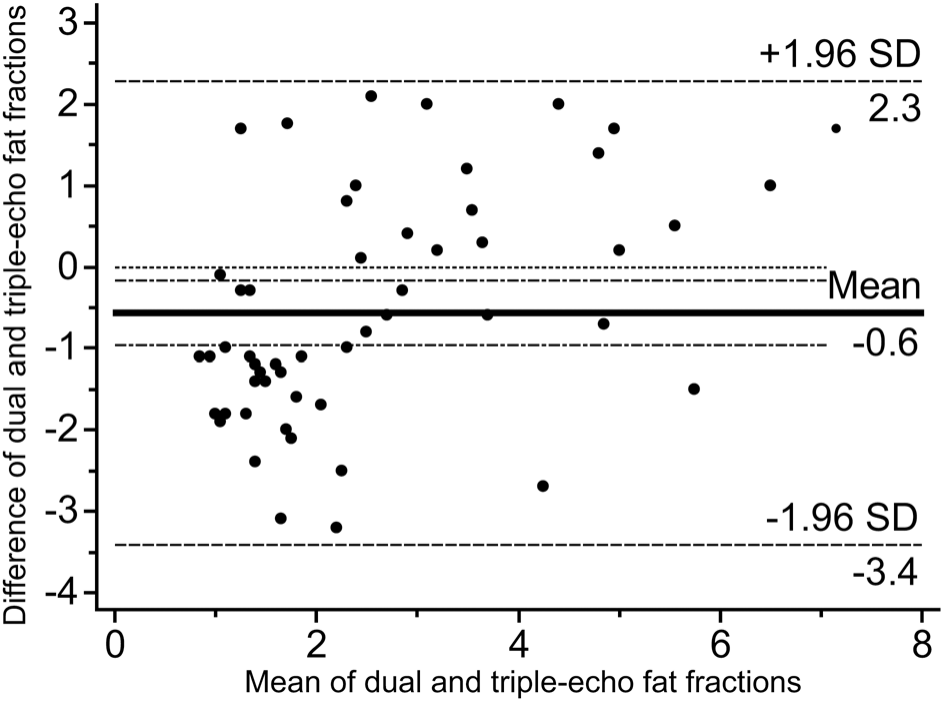
Bland-Altman plots for measurement of hepatic fat fraction using dual and triple-echo MRI. Bland-Altman plots demonstrated good agreement between dual and triple-echo fat fractions.

The median dual-echo fat fraction was 1.6% with a range of 0.1–8.0%, and the median triple-echo fat fraction was 2.7% with a range of 0.4–6.5%. The value of the dual-echo fat fraction was not significantly different from that of the triple-echo (*p* = 0.010) (Fig. 1). The Bland-Altman 95% limits of agreement were-3.4% to 2.2% for the difference between dual-echo and triple-echo fat fractions, compared with mean value of these two fat fractions (Fig. 2). In the Bland-Altman plot, all of the values were included within the range of 1.96 standard deviations of the difference, indicating significant agreement between dual and triple-echo fat fractions. There was also a tendency of differences concentrated below the mean value of-0.6% in small fat fractions (<3% fat fractions). This result implied that the triple-echo fat fraction tend to increase more than the dual-echo fat fraction when the fat fractions were small, 3% or less.

The results of the correlation analysis between clinical and laboratory findings and the hepatic fat fraction on DE- and TE-MRI are summarized in Table 1. There was no significant correlation between clinical and laboratory findings with dual- and triple-echo fat fractions. The serum triglyceride level and BMI showed a positive correlation with hepatic fat fraction without statistical significance. The serum cholesterol level showed a negative correlation on DE-MRI, while it had a positive correlation on TE-MRI, although neither correlation was statistically significant.

**Table 1:** Correlation between clinical and laboratory findings and hepatic fat fraction in dual- and triple-echo fat quantification MRI.

Parameters	Dual-echo fat fraction		Triple-echo fat fraction	
	r	*P* value*	r	*P* value*
Age (years)	-0.097	0.485	-0.087	0.530
Height (cm)	-0.128	0.355	-0.115	0.409
Weight (kg)	-0.009	0.951	-0.006	0.968
Body mass index (kg/m^2^)	0.195	0.159	0.206	0.136
Glucose (mg/dL)	-0.163	0.238	-0.155	0.262
Cholesterol (mg/dL)	-0.085	0.541	0.051	0.717
Triglycerides (mg/dL)	0.189	0.171	0.081	0.561

*from Spearman’s correlation test

The reference intervals were 5.9% (confidence interval 4.7–7.2) for DE-MRI and 5.6% (confidence interval 4.6–6.7) for TE-MRI. When using a known cut-off value of 5% fat fraction for fatty liver evaluation, eight children (8 of 54, 14.8%) on DE-MRI and six children (6 of 54, 11.1%) on TE-MRI showed a greater than 5% fat fraction, which can lead to a false positive diagnosis. Among the eight children with a greater than a 5% fat fraction on DE-MRI, six children had a 5–6% hepatic fat fraction and the remaining two had a 7–8% hepatic fat fraction. On TE-MRI, four out of six children had a 5–6% hepatic fat fraction, and two children had a 6–7% hepatic fat fraction. There were fewer healthy children who had more than a 6% fat fraction on both DE- and TE-MRI, than those who were above the 5% cut-off value. When we applied the cut-off value of a 6% fat fraction, as the nearest whole number from the reference interval, the false positive rate decreased to 3.7% (2 of 54) for both DE- and TE-MRI.

## Discussion

Prior studies have revealed that NAFLD is not an uncommon problem in children, with an estimated incidence of approximately 3–17% in American children and 38% in overweight and obese children [3,4,19]. Beyond its high prevalence, NAFLD is important as it can lead to more serious problems such as hepatic cirrhosis or hepatocellular carcinoma [3,19]. In addition, it can accompany metabolic syndrome, cardiovascular disease, or diabetes mellitus; however, it is reversible with early correction [3,19]. Therefore the early, accurate, and quantitative diagnosis of fatty change of the liver has very important clinical implications, especially for children.

Although the gold standard for diagnosis remains liver biopsy, this invasive procedure is not feasible in daily clinical practice and can raise ethical issues, especially when performed for screening healthy children for fatty liver disease. Therefore, recent studies have dedicated efforts to diagnosing fatty liver using ultrasound, CT, and MRI, including MRS. From these, DE-MRI has been used for a long time given its advantage of quantifying the hepatic fat fraction in the entire liver non-invasively [15,20,21]. Moreover, TE-MRI has improved the measurement of hepatic fat content by allowing a simple correction for T2* decay [11].

In previous reports, fatty change of the liver was defined as a greater than 5% accumulation of triglycerides in hepatocytes on histopathologic analysis [13,14]. It is well known that fat quantification MRI shows a strong correlation with hepatic steatosis level, especially in the assessment of macrovesicular steatosis, which is a common form of steatosis in NAFLD [5,22]. A recent study revealed that a cut-off value of 6.9% on triple-echo fat fraction MRI provided 93% sensitivity and 100% specificity in diagnosing hepatic steatosis in an adult population [23]; however, few studies on the normal hepatic fat fraction have been conducted in children. Fishbein et al. [24] applied a cut-off value of 9% to evaluate fatty liver in children using their previous results, using a value of greater than 2 standard deviations above the mean hepatic fat fraction of healthy adults. It is crucial to recognize the possibility of a difference between the normal ranges of hepatic fat fraction of children and adults on MRI. There was only one report that investigated the normal hepatic fat fraction in children. In 2011, Pacifico et al. [5] suggested a cut-off value of 4.85% for the diagnosis of fatty liver in children on MRI using a modification of the Dixon method; however, the standard hepatic fat fraction value in healthy children on DE- and TE-MRI is not yet well known, even though it is a basic sequence in almost all MRI machines.

Our study revealed the normal hepatic fat fraction ranges on DE-MRI (0.1–8.0%) and TE-MRI (0.4–6.5%) with a median value of 1.6% on DE-MRI and 2.7% on TE-MRI, and fat fractions using DE- and TE-MRI showed good agreement using Bland Altman plot. When we used a cut-off value of 5% for diagnosing hepatic steatosis, which is widely accepted as the normal range of hepatic fat fraction in histopathology, 14.8% of children (8 of 54) on DE-MRI and 11.1% of children (6 of 54) on TE-MRI had a greater than 5% fat fraction, which can result in false positive diagnosis. When we used a cut-off value of 6% for diagnosing hepatic steatosis, according to the reference interval, the false positive rate decreased to 3.7% (2 of 54) on both DE- and TE-MRI. Therefore, we suggest a cut-off value of 6% is more appropriate for children to diagnose fatty liver disease on both DE- and TE-MRI.

Our study also demonstrated that the triple-echo fat fraction showed a relatively narrow range (range 0.4–6.5%; median 2.7%) than the dual-echo fat fraction (range 0.1–8.0%; median 1.6%); however, we could not demonstrate a statistical difference in range. These results may be due to the high accuracy of TE-MRI for diagnosing fat fraction by minimizing the effect of iron deposition or inflammation from hepatitis, NAFLD, cirrhosis or hemochromatosis, as previously reported [11,25]. TE-MRI could reduce the susceptibility effect of iron by reducing T2* decay using a corrected IP, while the confounding T2 and T2* effect could result in uncorrected fat quantification on DE-MRI [7,26]. However, our study included only healthy children, and we cannot be sure of the effect of iron deposition in normal liver. Further studies regarding the accuracy of TE-MRI in pediatric liver disease are required.

In the analysis of correlation between the hepatic fat fraction on MR and clinical and laboratory findings, there was no significant correlation between clinical and laboratory findings with fat fractions. Previous reports suggested significant correlations between fat fraction on DE- and TE-MRI with BMI, gender, glucose, cholesterol, and triglyceride levels [5,16,17]. In a recent study of a pediatric cohort, Lee et al. [27] also revealed a strong correlation of hepatic steatosis and clinical data in pediatric patients. This difference could be from the different subject group of our study, as we included only healthy children. Lee et al. [27] explained that pediatric patients showed a stronger correlation of hepatic steatosis with clinical data than adults, due to low exposure to comorbidities and medication usage in children. Additional studies including not only healthy children but also pediatric fatty liver disease patients and overweight children are needed to evaluate the usefulness of DE- and TE-MRI in monitoring triglyceride level and detecting fatty liver more sensitively.

Our study has several limitations. First, we did not obtain histopathologic results of the liver as a reference standard. We presumed that children with normal ranges of clinical and laboratory parameters would have a normal hepatic fat fraction. Moreover, we did not perform follow-up in these children. Routine follow-up was not recommended in healthy children. However, there is a possibility of hidden hepatic disease in the enrolled children, and even liver biopsy also has limitations in evaluating diffuse liver disease, due to the heterogeneity of disease distribution in the liver [28]. Second, the number of included children was also small, given that abdominal MRI is not a commonly performed study during pediatric health maintenance visits. Additional studies with a large number of children and more concrete reference standards are required. The third limitation is that the image analysis using the region of interest was not obtained from the whole liver on both DE- and TE-MRI. This could affect the results of hepatic fat fraction depending on the position of the ROI. However, the measurement of ROI in the whole liver could result in an inaccurate fat fraction by including other anatomic structures such as vessels and bile ducts. The respiratory motion artifact in the liver dome as well as the partial volume effect also could affect the accuracy of the data. Therefore, we attempted to measure fat fraction using three ROIs in each image in the homogeneous parenchyma avoiding artifacts and vascular structures, as a representation of the whole liver in children.

In conclusion, the median value of the normal hepatic fat fraction was 1.6% (range 0.1–8.0%) on dual-echo sequences and 2.7% (range 0.4–6.5%) on triple-echo sequences with no differences among healthy children. We suggest a cut-off value of 6% fat fraction is more appropriate for diagnosing fatty liver on both dual- and triple-echo sequences as it can lower the false positive rate to 3.7% in children.
